# Inaugural Dissertation on Cancer of the Lungs

**Published:** 1843-04

**Authors:** 


Art. XI.

1. *Diss. Med. Inauguralis de Cancro Pulmonum.* Auct. E. N. Van Kleffens.—*Groningæ*, 1841. 8vo, pp. 72.
*Inaugural Dissertation on Cancer of the Lungs.* By E. N. Van Kleffens.—*Groningen*, 1841.

2. *Cases of Malignant Disease of the Lung.* By H. M. Hughes, m.d. (*Guy's Hospital Reports*, No. XIII.—*October*, 1841.)

3. *Clinical Lecture on Cancer of the Lung, delivered at University College Hospital.* By Dr. Taylor. (*Lancet, March* 26, 1842.)

4. *Researches on the Pathology and Diagnosis of Cancers of the Lung and Mediastinum.* By William Stokes, m.d. (*Dublin Journal of Medical Science, May*, 1842.)

5. *The Physical Diagnosis of Diseases of the Lungs*, (*Chapter relating to Cancer of the Lungs.*) By W. H. Walshe, m.d. 1843.

Did space permit, we should almost be tempted by the present favorable opportunity to enter into a complete examination of cancer of the lung in its anatomical, pathological, and practical relations. We should be justified in this course by the increasing interest of the subject, and above all by the obvious fact that, while its frequency, as a pulmonary affection productive of symptoms, is much greater than had until of very late years been suspected, it may at the bedside be confounded with diseases from which it is all-important to distinguish it. Unless a most careful inquiry into symptoms local and general, and more especially into the existing physical signs, be instituted, nothing is more likely than that cancerous solidification should be mistaken during life for tuberculous accumulation; nay more, that the error should remain uncorrected even at the post-mortem examination, unless the anatomical examination be most cautiously proceeded with. But for so full an inquiry as that alluded to, our means are insufficient, and we must therefore content ourselves with a more sketchy view of the present state of knowledge on the subject.

Of the three distinct species of cancer—Encephaloid, Scirrhus, and Colloid—which are now admitted to exist, the two former only have been satisfactorily detected in the lung. Among Dr. Hughes's cases is one, we are aware, in which some notion appears to have arisen in the mind of that writer that he had a mass of pulmonary colloid before him; but he himself admits "there will probably exist considerable difference of opinion upon the true nature of the case," and his description leads us in our own persons to realize the prediction. Colloid of the lung we, therefore, set down as among the anatomical changes of this viscus which remain to be observed. Encephaloid, on the other hand, is of much more common occurrence than scirrhus.

Dr. Stokes, speaking of the different forms of the disease, enumerates the following as having fallen under his observation:

"1. Isolated and generally well-defined encephaloid tubercles of a rounded form, the intervening tissue healthy, and the tumours equally distributed through both lungs. 2. Isolated masses of irregular forms; sometimes coinciding with a mass of complete cancerous degeneration. 3. Tubercles of various species of cancer coexisting, such as scirrhus, the encephaloid and the black spongiform cancer. 4. Simple degeneration of the whole or part of a lung into the homogeneous encephaloid matter. 5. Encephaloid tumours of the posterior mediastinum compressing the lung. 6 The same condition combined with cancerous degeneration and cancerous tubercles of the lung itself. 7. Cancerous tumour of the anterior mediastinum. 8. Tumours of white fluid cancerous matter perfectly encysted, and surrounding the trachea and œsophagus, combined with a white cancerous infiltration of a portion of the lung and cancerous coagula of the bronchial tubes. 9. Cancerous degeneration of the whole lung, with deep-seated and superficial ulcerous action, extensively separating the lung from the pulmonary pleura." (Dublin Journal.)

Upon some of these forms of the disease we proceed to offer a few observations.

The first form is that essentially characteristic of secondary cancer not only in the lungs but, as noticed by Cruveilhier, Dr. Walshe, and others, in the liver and bones. These tuberous disseminated masses almost invariably exist in the pulmonary substance, without giving rise to distinct symptoms during life, a fact so generally recognized by writers upon cancerous disease that it strikes us as rather singular to find Dr. Hughes commenting upon his experience to this effect as having some claim to novelty, on the plea that Dr. Stokes out of two or three cases did not meet with the variety in question. The immediate reasons that this form of cancer is unattended with obvious local symptoms appear to be the common existence of severe disease in other parts, acting as a sort of counter-irritant; and the peculiarly healthy state of the pulmonary tissue around and between the nodular morbid masses. Upon what this freedom from disease of the surrounding tissue depends, it is not easy to determine; it adds considerably to the difficulty of detecting the tuberous masses during life.

Dr. Stokes's fourth form of simple degeneration of the pulmonary substance is one which can rather be imagined than understood from the description given by himself, by Drs. Graves, Heyfelder, or Hughes. When Dr. Graves, for example, states that "in place of the right lung was found a solid mass, weighing more than six pounds; it was firm, homogeneous, and its substance resembled that of brain partly hardened by artificial means,"—he gives us no evidence that the pulmonary tissue had actually undergone a process of degeneration and not simply receded before a mass growing external to itself, and above all leaves us utterly in the dark as to the mode of development and advancement of interstitially infiltrated cancerous matter, together with the anatomical appearances of the structure thus diseased. Dr. Carswell's inimitable delineations, which leave so few points in morbid anatomy untouched, and, may we add, unadorned, yet throw no light on the condition at present in question; because, although he has distinctly figured infiltrated cancerous disease of the lung, the morbid matter so predominated over the healthy in the specimen employed, that no idea of the condition of the tissue while undergoing the process of infiltration is obtained from the plate. The case upon which Dr. Taylor's lecture is founded supplies this deficiency in a tolerably satisfactory manner, and we shall extract some portions of the description given by the lecturer:

"The colour of the implicated lung was a dirty, dark-greenish, brown; the texture hardly anywhere crepitant, exceedingly lacerable, yielding to the slightest traction or pressure; some portions had a granular aspect, others were broken down into a dark-coloured, dirty-looking, semi-fluid pulp; when cut or torn, in most parts the cut surface was speedily covered with a similar fluid; the odour was offensive, but not excessively so; a foreign deposit was copiously diffused throughout the substance of nearly the whole lung; in most places it was in small masses, of a dirty-gray colour, and very much resembling diffused crude tubercle, which it was supposed to be."

So far the description, though not fairly applicable to ordinary tuberculization, but apparently referrible in some part to gangrenous tissue, yet portrays none of the characteristic features of cancerous deposition; we are, however, informed further on that, "whilst examining the matter infiltrated through the tissue of the lung, Dr. Walshe observed that its surface in various places had a pink hue and was distinctly vascular, proving that it was not tubercle, and he subsequently ascertained its cancerous nature beyond all doubt by microscopical examination." It may be well to add, that there was in this case "about the root of the lung, a considerable quantity of encephaloid tissue; rather firm, of a brain-white colour, mixed with black portions (apparently the remains of bronchial glands,) and when compressed, a creamy fluid exuded from numerous points of its cut surface."

Respecting the eighth form of disease referred to by Dr. Stokes, we can only say that the extreme rarity of encysted cancer, either in the lungs or elsewhere, would have justified him in entering into a full description of the anatomical features of the morbid accumulation.

The rarity of ulceration of cancer of the lung is sufficiently attested by the fact that two cases, one observed by Dr. Taylor, the other by Dr. Stokes, furnish the only evidence of the fact as far as we know, to be found on record. In the curious instance described by Dr. Stokes, the whole organ was converted into a mass, having less consistence than common in encephaloid disease; a large portion of the lung was burrowed by anfractuous excavations, communicating on the one hand with the bronchial tubes, and on the other terminating in fistulæ, running in various directions to the surface of the lung, where they terminated in superficial cavities (containing air and a whitish purulent fluid,) bounded at opposite points by the posterior surface of the pulmonary pleura, and by the degenerated pulmonary substance. The left lung is said to have been healthy, except that it contained "a few small hydatids." The term hydatid has been so vaguely applied that we wish Dr. Stokes had distinctly signified the species of formation to which *he* applies the term; if he mean by the word real acephalocysts, the case acquires new interest, for it would appear from the inquiries of Dr. Walshe, (Cyclopædia of Surgery, vol. i. p. 616,) that the association of cancer and these entozoa is extraordinarily rare.

Cancer occurs in either lung only, or in both simultaneously; of nineteen cases collected by Kleffens six were examples of cancer of the right lung, seven of disease of the left, while in the remaining six both organs were implicated. But this calculation refers apparently to cases of all kinds, those in which the pulmonary affection was primary, and those where in it constituted a part only of a general cancerous diathesis. If secondary cases were excluded from consideration, (those cases in which the tuberous masses form the anatomical condition of the disease) we believe that the proportion of *double* cases would be much lower. Indeed in the instance of the disease which came under Dr. Taylor's notice, the absence of disease in one lung, while the other was affected to a very considerable amount had its influence in leading that physician to consider the case one of cancer and not of tubercles. Dr. Hughes, with some hastiness, for he has but four cases whereon to ground the opinion, considers the right lung especially prone to the disease ; although premature, the conclusion may be a correct one, however, for several other cases might be added to the four in question : Dr. Sims published two; Dr. Taylor's case, that of Dr. Graves, and two by Dr. Stokes, furnish examples of the same localization, and Dr. Walshe briefly alludes to another. (p. 285.)

Among twenty-seven cases collected by Van Kleffens, twenty-two were instances of the coexistence of cancer in two or more different organs: in fifteen of those twenty-two the disease existed in external parts, a circumstance which of course, as has been noted by all writers on the affection, removes much of the difficulty of the diagnosis. Van Kleffens does not state in how many of these cases the pulmonary cancer was primary.

Females are probably more prone to the disease than males. Cancer in general is an affection said to be about three times as frequent in females as in males,* and may therefore be supposed to affect the lungs more frequently in them as well as other parts; nevertheless Van Kleffens found that of twenty cases, thirteen were furnished by males and seven by females. The affection has been observed at all ages from twenty to seventy; but appears to be most common in individuals aged between twenty and thirty, a circumstance which agrees with what has been already said respecting the greater frequency of the encephaloid species of the disease.

Van Kleffens has analysed the symptoms in a number of cases, varying in different instances from twelve to seventeen. In ten cases cough, he states, is distinctly mentioned ; it is more probable that it was forgotten by the reporters than absent in the others. The cough is said to have been dry in two of these cases; the matter expectorated in the others varied: mucous and bloody in some ; in a few mucous only or purulent; in not very rare instances there was actual hemoptysis. Dr. Hughes lays stress on the circumstance of the sputa "occasionally consisting of blood, so thoroughly incorporated with serous fluid as to resemble *red* currant jelly ;" while Dr. Stokes compares the expectorated matter to *black* currant jelly. Dr. Taylor's patient had had hemoptysis ; but swallowed her sputa while under observation ; the breath was exceedingly *fetid* ; and a similar state of the sputa, so marked as to require the constant use of chlorine, existed in a patient whose case has been related by Canstatt. (Hannov. Annalen, Bd. v. Heft 3, S. 433, 1840.)

Dyspnea, persistent or occurring in paroxysms, has been noted in almost every case on record, and unless in cases of disseminated masses through the lung with a healthy state of the intervening tissue might *à priori* be expected to exist in every instance, Yet that it may be completely absent, neither attracting the attention of the medical attendant,

* The proportion is, however, not precisely this ; at least, according to the calculation of Dr. Walshe, females in England suffer only two and a half times as frequently as males. (Cyclopædia of Surgery, vol. i. p. 626.)

nor exciting complaint on the part of the patient, is sufficiently proved by Dr. Taylor's case. But we cannot think this physician justified in attaching any importance to such absence of dyspnea as distinguishing the disease from pleuritic effusion. As his own language shows him to be aware, dyspnea may be totally absent in cases of very considerable liquid collection; and we venture to affirm that it will be more frequently absent in such cases than in those of cancerous infiltration.

The subject already referred to, who came under the observation of Canstatt, suffered no pain; in the great majority of instances this symptom is noted, in some of them described as severe and darting, in others as associated with pain in the shoulder of the affected side; in one case (recorded by Heyfelder,) assimilated to electric darts passing from the false ribs and sternum to the spine. Dr. Stokes enumerates among the symptoms "important as indicative of this disease, pain of a continued kind," and elsewhere speaks of his inclining to the diagnosis of cancer in a case under his observation, in consequence of the patient's suffering under "a violent attack of pain in the side, the pulse remaining natural."

Facts are wanting to determine whether the alleged *characteristic* sputa may in reality be considered to have this signification. As to the other symptoms above briefly passed in review, their absolute want of diagnostic importance needs not to be insisted upon. In respect of symptomatology, indeed, the cases hitherto published are with rare exceptions singularly imperfect; some of those reprinted by Van Kleffens are in the happiest "old school" style of laconic unmeaningness.

With physical signs the case is fortunately different. There are few affections of which the auscultatory and other physical evidence has been established, upon so short an acquaintance with the disease, with any approach to similar accuracy, a sufficient proof that these signs must in their modes of succession or combination, if not in their nature, possess some tolerably distinctive peculiarities. And this is not a matter of book-learning; the affection has been successfully diagnosticated in not a few instances, and with each new case almost some additional particulars, of a kind to facilitate its future diagnosis, have been ascertained. In examining this part of the subject we shall take the synoptical view of the signs of cancer in Dr. Walshe's recent work as our guide, and in our comments thereon, avail ourselves of several of the observations of that writer, of Dr. Stokes, and of Dr. Taylor.

There is an important distinction to be established between different cases of cancer, derived from the condition of the walls of the chest. This distinction was, we believe, first seized by Dr. Stokes, and depends upon the existence or absence of dilatation of the affected side, while this in its turn furnishes some clue to the form in which the cancerous matter is deposited. Dilatation, in truth, never occurs when the cancerous substance is infiltrated through the pulmonary tissue: but may be absent or present in cases of the tuberous form according as the masses accumulated are of limited or of considerable bulk. We have thus one class of cases of "infiltrated cancer of the lung; alone or combined with tuberous cancer, but not to such extent, as to cause dilatation of the side;" and a second of cases of "tuberous cancer of the lung or pleura, with dilatation of the side." Of the first class Dr. Walshe enumerates the signs as follows. Signs ascertained by

"INSPECTION: *Retraction of the affected side; diminished motions of expansion and of elevation; diminished costal motions;* intercostal spaces deeper than natural. APPLICATION OF THE HAND: *Vocal and tussive vibration diminished in intensity.* MENSURATION: *Semicircular measurement diminished;* deficient increase of width during inspiration. PERCUSSION: *Sound intensely dull,* and of short duration; *resistance of walls marked;* special character of sound tubular in some cases about the edges of the infra-clavicular and also the mammary regions; the limits of the dull sound natural beyond the middle line in some cases."

So far the signs apply to infiltrated cancer previous to, or after the occurrence of softening; the remaining signs differ according as this change has or has not set in.

"(*Before softening of the cancerous matter.*) AUSCULTATION: *Respiration of diffused blowing type strongly masked,* [marked?] or with progress of disease (which leads to obstruction or obliteration of the bronchi,) *weak or almost suppressed,* retaining, as long as it exists, its blowing or bronchial character; *respiration exaggerated on healthy side; bronchophony; bronchial cough; heart's sounds transmitted with increased intensity.* (After softening of the cancerous matter.) PERCUSSION: *Sound may become somewhat clearer, and the resistance of the walls diminished.* AUSCULTATION: *Cavernous respiration; mucous, cavernulous or cavernous ronchus.* SITUATION OF SURROUNDING PARTS: *Mediastinum,* more rarely the heart, *detruded to the opposite side; corresponding division of the diaphragm, with its subjacent viscera, may be depressed.*" (p. 140.)

Retraction of the affected side is a sign of considerable importance; in itself alone it would be enough to distinguish a case of cancerous infiltration from pleurisy with effusion in its earliest stages. Pleurisy of longer duration, in which the process of absorption had made some progress, and a diminution of bulk of the side become slightly apparent, might bear some resemblance to the case in question; but it is remarked by Dr. Taylor that "many of the intercostal spaces would generally be obliterated" in the pleuritic affection, whereas they are more marked than natural in the cancerous; the signs derived from inspection would consequently suffice to distinguish the cases, were it necessary to limit our investigation to those signs.

In Dr. Walshe's enumeration of the signs appears "*diminished* vibratile tremor of the voice," and the majority of published cases, especially that of Dr. Taylor, the most precise yet recorded, bear witness to his correctness in including it among them. Indeed there is only one case before us in which an increase of tactile vibration is said to have been perceptible, and here some anatomical peculiarity probably existed to account for the exceptional occurrence. We are among those, however, who believe that vocal vibration is a sign often fallacious and seldom of real importance: a very similar opinion is expressed by Dr. Walshe in the work before us, warranted, he states, by his own experience and certainly, beside this, by the marked contradiction he has shown to exist in the doctrines taught by various writers respecting it.

The intensity of the dull sound over lungs affected with cancerous degeneration appears to have impressed itself more or less strongly upon all these authors; and two of them have particularly commented upon the extremely marked sense of resistance experienced by the finger on percussion. Dr. Walshe has found this so great that it was "almost painful to use the finger as a pleximeter;" Dr. Taylor seems to consider it greater than could occur in pleurisy, and therefore not without its utility in distinguishing the two species of disease. But one of the conditions of the sound elicited by percussion in Dr. Taylor's case resembled very closely, or was identical with that observed in certain cases of pleurisy, with contraction of the side, the resonance was amphoric, metallic, and like that produced by striking an empty bottle with an open mouth. Our attention has recently been arrested in two or three cases of chronic pleurisy by this condition of the sound of percussion ; and although sufficiently analogous to the tympanitic, yet it cannot by an attentive observer, at least when put upon his guard, be confounded with that description of resonance. The same phenomenon is observed in certain cases of pneumonia, and depends upon the bronchial sound of percussion being transmitted, unaltered, by the consolidated pulmonary substance ; such at least is by some admitted to be the true explanation, although we find that Dr. Stokes advocates the notion of its depending upon effusion of air into the pleura.

The conditions of the respiratory murmurs in this disease are remarkable. At first possessing a most strongly marked bronchial, tubular or blowing character, they have been observed to become gradually weaker and weaker with the progress of the disease. The explanation of this increasing weakness tendered by several of these authors is that diminution of the caliber of the larger bronchial tube leading to the part interferes with the entry of air ; in a case by Dr. Graves this change would appear to have afforded an insurmountable obstacle to respiration, for we are informed "the tracheal respiration in the front of the lung soon *disappeared.*" The bronchial respiration appears to be in itself an important sign, as its intensity is stated to be much more marked than in any other affection except pneumonia.

The existence of strong puerile respiration in the unaffected lung, (when one only was implicated,) has been noted in several instances.

After softening has commenced in the cancerous matter, the auscultatory signs may be supposed, necessarily to undergo change. But experience so far permits us scarcely to venture any very fixed opinion as to the nature of the new phenomena. Dr. Taylor's and one of Dr. Stokes's cases appear to be the only ones on record in which they were carefully reported ; but they must have much more frequently been observed, although their real cause had not been established. The signs are those of softening of tubercle, liquid ronchus assuming the cavernous characters and cavernous respiration as the excavation proceeds. It is fortunate for the facility of diagnosis that ulceration is not common in these cases ; or at least that it is likely seldom to have set in before the patient submits himself to medical treatment. And for this reason, the combined physical signs under this condition of the disease bear a much stronger similarity to those of tubercle than previous to ulceration ; Dr. Taylor in framing his opinion respecting the nature of the affection before him, (this was before any signs attributable to softening had become audible,) inclined to the diagnosis of cancer, because, among other reasons, "if the whole lung were condensed by tubercles, some of them would probably have begun to soften and given rise to more or less muco-crepitant ronchus."

It will follow from what has been said that the thoracic ordinary physical signs of this disease are at the least tolerably distinctive ; there are some other physical conditions indicative of tumour, commonly to be detected, and when these, besides the phenomena already described, exist, there can be no doubt of the nature of the malady. They are stated as follows by Dr. Walshe :

"1, Enlargement and extreme congestion of the jugular, axillary, mammary and superior epigastric veins ; 2, A notable difference in point of fulness between the two radial pulses ; 3, Œdema of the affected side, corresponding arm and side of the face ; 4, Fulness of the neck ; 5, Prominence of the eyeballs, which, combined with the condition of the neck and face, gives the patient the appearance to a certain extent of a strangled person ; 6, Dysphagia ; 7, The existence of tumours in other parts of the body."

All these appearances (except the last) clearly depend upon venous obstruction, and will be present or absent according as such obstruction does or does not exist : in Dr. Taylor's case they were absent. The growth of tumours in other parts of the body has been of great service in diagnosis in many cases. "One of the most interesting circumstances in this case," says Dr. Stokes, in commenting on a narrative by Heyfelder, "was the growth of the external tumour during the last period of the patient's illness ;" a similar phenomenon was observed in the case published by Dr. Graves, in which, towards the close of the disease, three tumours appeared, and increased with great rapidity.

It is observed by Dr. Stokes that in the advanced periods of the disease, as in aneurism, gangrene of a portion of the lung may supervene. Dr. Stokes adds that it has been shown by Dr. Macdonnell that from the anatomical disposition of the arteries of the lung, pressure upon any part of the main bronchus might cause the death of the lung ; of course, as is observed by this writer, the liability to this is greater in the case of mediastinal tumours than in the simple degeneration.

We have next to consider the signs of "tuberous cancer of the lung or pleura with dilatation of the side :" we shall again extract from Dr. Walshe's volume.

"INSPECTION : *Affected side, expanded or affected with bulging inferiorly ; intercostal spaces widened, flat or even convex ; motions, both general and costal, abolished completely ; fluctuation never visible in intercostal spaces.* APPLICATION OF THE HAND : *Surface unnaturally smooth and even ; vocal and tussive vibration abolished ; neither simple fluctuation* nor peripheric fluctuation *to be detected ; pulsatile vibration sometimes present.* MENSURATION : *Increase of semi-circular measurement of the side ;* width of side unaltered by respiration ; *antero-posterior diameter increased ; vertical measurement increased ;* distance between the nipple and median line greater than on the opposite side. PERCUSSION : *Sound completely and most extensively dull and of short duration ; resistance of walls extreme ; limits of the dull sound not altered by changing the position of the patient.* AUSCULTATION : *Respiration of the diffused or tubular blowing type, intensely developed in some cases ; ronchi either absent or those of co-existing bronchitis ; bronchophony sometimes so intense as to amount almost to pectoriloquy ; bronchial cough ;* heart's sounds transmitted with unnatural intensity ; double additional pulsation with blowing murmurs sometimes heard also ; exaggerated respiration on the healthy side. SITUATION OF SURROUNDING PARTS : *Heart and mediastinum detruded to the opposite side ; corresponding division of the diaphragm depressed with its subjacent viscera,* sometimes to a very great amount." (p. 141.)

The general similarity of the signs here displayed to those attending pleurisy, with a large amount of effusion into the chest, will not fail to strike the reader. Nor is it wonderful that this similarity should exist, so closely analogous are many of the circumstances of the two cases.

But the nature of the materials causing dilatation differs; in one instance they are fluid, in the other solid, and hence are derived some peculiarities in the signs of each affection. Thus, as Dr. Walshe observes, simple fluctuation in the pleura will be invariably absent, and "in all probability, even in cases of the most diffluent encephaloid, *peripheric fluctuation*" similarly wanting. With this last-named phenomenon, as a sign of pleuritic effusion we confess ourselves hardly familiar; but from the description of the manner in which it is to be obtained, we are inclined to admit that it will not be detected in encephaloid disease, however softened the mass may be. At present, however, we agree with Dr. Walshe in the propriety of making a question of its invariable absence. The resistance of the walls under percussion will be more intensely marked when the morbid accumulation is solid than liquid; upon this point we have already spoken. The blowing respiration heard in cancerous cases will always far exceed, both in intensity and in extent, that sometimes audible in cases of fluid collection; such at least appears to be the opinion of several of these authors, and we perceive no reasonable ground of dissent. Dr. Walshe insists also upon the intensity of the bronchophony commonly discovered, and the total absence of ægophony; we must confess that, although this observer is justified by the records of some few cases in admitting the existence of ægophony in rare cases of *abundant* pleuritic effusion, we consider such cases too purely exceptional to be taken into account. The unnatural distinctness with which the heart's sounds are transmitted, and occasionally the existence of pulsation in the lung, as in cases of Dr. Sims and Stokes, will tend further to guide the physician in his diagnosis. This pulsation is however by no means a constant occurrence in cases of carcinomatous tumour. Dr. Stokes, who drew the attention of observers to it, was doubtful whether in the instance met with by him, "the pulsation was from the vessels of the cerebriform mass, or communicated by the pulmonary artery." He states that he strongly inclines to the latter opinion; yet the vessel seems scarcely to have been in a condition to have produced such pulsation, "the trunk of the pulmonary artery was compressed and flattened, its section presented an elliptical form, and its caliber was so much diminished as to admit only a full sized catheter." (Diagnosis and Treatment of Diseases of the Chest, p. 379.) In addition to the differences now passed in review, it must be remembered that in many cases of cancer, the signs of compression of the thoracic vessels and of the œsophagus already enumerated are present; whereas, as far as experience goes, those signs are never produced in empyema, no matter how extensive.

Upon the treatment of this affection, authors have not yet spoken: their discretion is laudable.

Before leaving this subject we would direct the attention of our readers to one of Dr. Stokes's cases, that of "ulcerated cancer of the lung with extensive separation of the pleura;" we could not succeed in condensing it effectually and it is too long for extraction. Suffice it to say, that so extraordinary were the physical signs, that various persons who examined it, (Dr. Stokes himself "never ventured on giving a diagnosis,") conceived it successively to be phthisis; chronic pneumonia with ulceration; empyema and pneumo-thorax; hernia of the abdominal viscera through the diaphragm; and emphysema of the liver!

